# Investigating changes of functional brain networks in painful temporomandibular disorders: a resting-state fMRI study

**DOI:** 10.22514/jofph.2026.006

**Published:** 2026-01-12

**Authors:** Yujiao Jiang, Xin Li, Mengqi Liu, Zhiye Chen

**Affiliations:** ^1^Department of Radiology, Hainan Hospital of PLA General Hospital, 572013 Sanya, Hainan, China; ^2^School of Medical Imaging, Bengbu Medical University, 233030 Bengbu, Anhui, China; ^3^Department of Nuclear Medicine, Xinqiao Hospital, Army Medical University, 400037 Chongqing, China

**Keywords:** Temporomandibular disorders, Pain, Resting-state fMRI, Brain networks, Clustering coefficient, Local efficiency

## Abstract

**Background**: Temporomandibular disorders (TMD), particularly pain-related TMD (TMDp), are closely associated with social and psychological factors. However, the neuromechanisms of pain of TMDp are still currently unclear. This study aimed to investigate the altered topological properties of the brain network in the TMDp patients using resting-state functional magnetic resonance imaging (rs-fMRI), and to explore the association between these parameters and emotional and clinical variables of TMDp. **Methods**: A total of 41 TMDp patients and 33 age- and gender-matched healthy controls (NCs) were recruited, and rs-fMRI data were obtained from a 3.0T MR scanner. The topological properties of brain functional networks were calculated based on the rs-fMRI data and were compared between two groups to investigate the altered topological characteristics in TMDp. The correlation analysis was also performed between the abnormal topological characteristics and the clinical variables in TMDp patients. **Results**: TMDp patients presented significantly decreased clustering coefficient (Cp) and decreased local efficiency (Eloc) when sparsity threshold was 0.05 and 0.06 compared with NCs (*p * < 0.05), and the Eloc values when sparsity threshold was 0.06 were positively correlated with depressive (*r* = 0.319, *p* = 0.042) and anxious (*r* = 0.348, *p* = 0.026) variables in TMDp patients. **Conclusions**: The current study demonstrated the abnormal topological changes of the brain network were observed in TMDp, which could be helpful in understanding the neuromechanisms of pain of TMDp. The topological properties of the brain network based on rs-fMRI could be considered as a new simple tool to monitor the dysfunction network of the brain in TMDp.

## 1. Introduction

Temporomandibular disorders (TMDs) are defined as a complex clinical oral disease that are related to alterations in the structure, function, or physiology of the masticatory system [1, 2], and may be accompanied by other local symptoms or general discomfort, according to the American Academy of Orofacial Pain. While temporomandibular disorders affect nearly 28% of the general population, predominantly young women, the clinical course varies significantly. Only a minority (5–10%) of symptomatic patients require active treatment, with up to 40% experiencing natural symptom remission without intervention [3]. The global prevalence of temporomandibular disorders (TMD) exhibits significant geographical variation. In the United States, approximately 40–75% of adults report at least one TMD symptom, with chronic temporomandibular joint (TMJ) pain specifically ranging from 5% to 12% [4] and an annual incidence rate of first-onset TMJ pain approaching 4% [5]. A Finnish clinical study utilizing standardized diagnostic protocols found that 38% of participants presented at least one TMD sign, demonstrating notable gender disparities where women consistently showed higher prevalence rates. The most frequent clinical manifestations included TMJ clicking (15%) and masticatory muscle palpation pain (14%) [6]. Notably, adolescent populations worldwide show increasing TMD prevalence estimates ranging from 7% to 30% [4], with Brazilian epidemiological data revealing particularly high rates (33.2%) when applying the RDC/TMD Axis I diagnostic criteria [7].

TMDs can be accompanied by characteristic clinical signs and symptoms, such as oral-facial pain, headache, joint noise, irregular mandibular movement, and limitation of mouth opening. Temporomandibular joint pain is one of the most common symptoms in TMD. Typically, TMD-related pain is not severe but manifests as chronic pain—persisting for at least three months, recurring, and originating from tissues such as the joint, muscles, and/or fascia. It ranks as the third most common chronic pain condition, following tension-type headache and back pain [8, 9, 10]. Previous studies suggested that TMDs were a major influencing factor for non-dental orofacial chronic pain and are also related to chronic neck pain and headaches [4, 11]. Recent studies have shown that TMDs affect approximately 30% of the Chinese population with a higher incidence among younger individuals [12], however, the presence of clinical symptoms gradually increase at more advance ages [13]. The pathogenesis of TMD has not been fully clarified, is considered a comprehensive disease under the action of multiple factors, such as lifestyle and diet changes, lateral chewing, breathing habits, can lead to a significant increase in prevalence [14, 15]. Additionally, negative psychological factors such as anxiety, tension, depression, and sleep disorders also plays an important role in the development of the disease, is also an important factor affecting the severity of clinical symptoms in patients [16]. Given the high prevalence of TMDs and their substantial impact on patients’ quality of life, further research on pain-related TMD (TMDp) patients is required to address clinical needs [17].

Resting-state functional MRI (rs-fMRI) represents a non-invasive imaging modality that evaluates functional brain activity through monitoring spontaneous fluctuations in blood oxygen level-dependent (BOLD) signal intensity, thereby helping to explain the pathological and physiological changes in brain function. Many brain imaging studies have revealed alterations in brain function and structure of TMDp patients [18, 19, 20, 21], indicating that central nervous system (CNS) mechanisms contribute to symptom development or maintenance [22, 23], as well as the process of pain amplification and chronification. The rs-fMRI studies on chronic pain mainly used the functional connectivity (FC) method, in which the brain is treated as a network of interacting components, and have revealed brain connectivity alterations in pain patients with chronic lower back pain, complex regional pain syndrome, osteoarthritis and TMD [24, 25, 26]. Previous research has reported disruptions of functional connectivity between several pain-related brain regions, including corticostriatal networks (CN), the salience network (SN), and the default mode network (DMN) in TMDp patients, which probably resulting in deficits in motor control, pain processing, and cognition in TMD [25, 27, 28]. However, most of these studies have focused solely on alterations in the local brain structure and function.

As it is known that structural or functional abnormalities in specific brain regions can be mapped to the network level. Thus, analyzing abnormal changes across the entire brain functional network would provide deeper insights into the pathophysiological mechanisms underlying TMDp. In this study, we hypothesize that TMDp patients may present altered topological properties of the brain network. To address the hypothesis, we prospectively obtained the rs-fMRI data from 41 TMDp patients and 33 normal volunteers. We calculated and compared the topological attributes of their brain networks to identify topological changes, aiming to elucidate the neural mechanisms of temporomandibular joint pain in TMDp. Additionally, we performed correlation analyses between network attribute values and clinical symptoms. Overall, this study investigates the differences between TMDp patients and healthy individuals from the perspective of whole-brain functional networks, based on the topological properties of brain networks.

## 2. Materials and methods

### 2.1 Participants

The study protocol was approved by the Ethics Committee of the Hainan Hospital of PLA General Hospital (No. S2022-03) and was performed in accordance with the ethical guidelines of Declaration of Helsinki. Written informed consents were obtained from all participants. The inclusion criteria for patients with pain-related TMD were not taking prophylactic medication or anodyne for more than 10 days per month during the last 3 months.

The study cohort comprised 41 patients with TMDp and 33 demographically matched healthy controls (NCs), all recruited from the inpatient and outpatient departments of Hainan Hospital of PLA General Hospital during the post-September 2022 period. Inclusion criteria for TMDp patients included: (1) the patients diagnosed with TMDp were examined by a single dentist with experience in orofacial pain and a radiologist according to MRI results and the evidence-based Diagnostic Criteria for pain-related Temporomandibular Disorders (DC/TMD) [29, 30]; (2) full permanent dentition; (3) presence of pain in the face, masticatory musculature, or head under the close-mouth state or when active; (4) patients with pain symptoms for more than 3 months and without any TMD-related treatment; (5) not taking prophylactic medication. Excluded prophylactic medications included analgesics (*e.g.*, Non-Steroidal Anti-Inflammatory Drugs (NSAIDs), opioids), antidepressants (*e.g.*, Selective Serotonin Reuptake Inhibitors (SSRIs), Serotonin-Norepinephrine Reuptake Inhibitors (SNRIs)), anticonvulsants (*e.g.*, gabapentin), and muscle relaxants, administered >10 days/month over the preceding 3 months. This ensured homogeneity in neurophysiological baselines and minimized pharmacological confounders. Exclusion criteria for all participants included: (1) under 18 years of age or left-handed; (2) other TMJ diseases such as tumor, maxillofacial trauma or TMJ surgery history; (3) psychiatric disorders; (4) systemic or malignant conditions including diabetes, rheumatic disorders, and gout; (5) contraindications to MRI; (6) poor image quality. And the inclusion criteria for NCs included: (1) age 18–65 and right-handed; (2) without any clinical signs and symptoms of TMD or any pain.

All participants first completed a set of psychometric instruments assessing pain symptoms and mental health status before proceeding to the MRI examination. The clinical data of all subjects were evaluated using the Jaw Functional Limitation Scale, oral behavior checklist and neuropsychological assessments scale published on the DC/TMD International Union website (http://www.rdctmdinternational.org) in 2016, and the Hamilton Anxiety Scale (Hamilton anxiety scale, HAMA) and the Hamilton Depression Scale (Hamilton depression scale, HAMD) were added to assess the psychological status of the participants. The Generalized Anxiety Disorder-7 (GAD-7) questionnaire and Patient Health Questionnaire-9 (PHQ-9) were administered to systematically assess anxiety and depression levels, respectively, while somatization symptom severity was clinically assessed using the validated Patient Health Questionnaire-15 (PHQ-15). All subjects also completed Jaw Functional Limitation Scale (JFLS) for evaluation of functional situation of TMJ and Oral Behavior Checklist (OBC) for evaluation of oral bad behavior frequency. Hamilton Anxiety Scale (HAMA) and Hamilton Depression Scale (HAMD) were performed for clinical assessment of depression and anxiety for all individuals, and the Visual Analog Scale (VAS) was administered to evaluate subjective pain perception in the TMDp cohort.

### 2.2 MRI data acquisition

Brain imaging data were acquired on a 3.0T MR scanner (Ingenia CX, Philips Healthcare, Best, the Netherlands) using a 32-channel head coil. Subjects were positioned supine on the MRI scanning bed, and both sides of the head were fixed with sponge cushions in order to reduce head movement. All scans were acquired under standardized resting conditions-wakeful relaxation with eyes closed, controlled respiration, and strict motion avoidance. rs-fMRI data were obtained by the BOLD gradient echo echo-planar imaging (GRE-EPI). The parameters are as following: repetition time (TR) = 800 ms, echo time (TE) = 32 ms, acquisition matrix = 88 × 97, flip angle (FA) = 52°, field of view (FOV) = 210 mm × 236 mm, slice thickness = 2 mm, interval = 0 mm, number of excitations (NEX) = 1, voxel size = 2.4 mm × 2.4 mm × 2 mm, and scan time about 3 minutes. The resting state run produced 180 volumes, and the scan covered the whole cerebrum and cerebellum.

### 2.3 Data processing

We used BRAinNetome Toolkit [31] (BRANT, https://brant.brainnetome.org) for functional image preprocessing, which were run under MATLAB 2021b (The Mathworks Inc., Natick, MA, USA). The image processing steps are as follows: Functional preprocessing included: (1) digital imaging and communications in medicine (DICOM)-to-Neuroimaging Informatics Technology Initiative (NIFTI) conversion; (2) removal of first 10 volumes; (3) slice-timing correction; (4) realignment (motion threshold: 2 mm/2°); (5) Montreal Neurological Institute (MNI) spatial normalization (resampled 3 mm^3^); (6) band-pass filtering (0.01–0.08 Hz); (7) Gaussian smoothing (6 mm full width at half maximum (FWHM)).

### 2.4 Construction of brain network connection matrix

The brain network connection matrix was constructed on BRANT software, and the whole brain was divided into 90 different nodes based on the anatomical automatic labeling (AAL) template, and the average time series of each node was extracted separately. Pearson correlation analysis was carried out between pair-to-node with the negative correlations retained in network construction, and the functional connection matrix of the changed resting state was obtained.

### 2.5 Analyses of topological properties of the brain networks

In the study of topological properties of brain functional networks, the sparsity threshold ranged from 0.05 to 0.20 (step 0.01) to ensure that the constructed functional connection matrix can retain real and effective connections and avoid fragmented/overconnected networks. Then, we calculated the topology properties of each subject’s brain networks under each sparsity threshold, including clustering coefficient (Cp), global efficiency (Eg), local efficiency (Eloc), shortest path length (Lp), and small-world attributes including standardized clustering coefficient (γ), standardized shortest path length (λ), and small-world index (σ) to quantify efficient information transfer.

### 2.6 Statistical analysis

Statistical analysis of the clinic data was conducted using IBM SPSS software version 22.0 (IBM Corp., Armonk, NY, USA). Continuous data were analyzed based on their distribution: normally distributed measures were expressed as mean ± SD, while skewed variables were described with the median (the first quartile, the third quartile).

The Chi-square test was used to examine the differences in qualitative variables, and independent *t*-test was used to examine the differences in quantitative variables. Correlation analysis was performed between the global and node attribute indicators with significant differences and clinical variables. Pearson method was performed to evaluate the correlation between the normal distribution data, and Spearman method was performed to test for the correlation between the non-normal distribution data. *p * < 0.05 was considered to be a statistically significant difference.

## 3. Results

### 3.1 Demographic and clinical variables

Demography and clinical characteristics of 41 TMDp patients and 33 NCs were summarized in Table 1. There were no significant differences for the age, gender, HAMA, HAMD, GAD-7, PHQ-9, PHQ-15 or OBC scores (*p * > 0.05) between the two groups. The JFLS scores of TMDp patients (35.00 (50.00, 16.00)) were significantly higher than that of NCs (3.00 (6.50, 0.00)) (*p * < 0.05).

**Table 1.** Demographic and clinical characteristics of the subjects.

Clinical variables	TMD	NC	*p* value
Num (M/F)	41 (18/23)	33 (12/21)	0.511
Age^a^	25.00 (22.00, 33.00)	25.00 (23.00, 26.50)	0.785
HAMA^a^	4.00 (2.00, 6.00)	3.00 (1.00, 6.50)	0.450
HAMD^a^	3.00 (1.00, 5.00)	2.00 (1.00, 4.00)	0.305
GAD-7^a^	3.00 (0.00, 7.50)	1.00 (0.00, 5.00)	0.157
PHQ-9^a^	4.00 (1.00, 8.50)	4.00 (2.00, 7.00)	0.710
PHQ-15^a^	3.00 (0.50, 8.50)	3.00 (0.00, 5.00)	0.353
JFLS^a^	35.00 (16.00, 50.00)	3.00 (0.00, 6.50)	<0.001
OBC^a^	18.00 (13.50, 24.50)	15.00 (11.50, 26.00)	0.439
VAS^a^	3.00 (2.00, 4.50)	NA	NA

*HAMA, Hamilton Anxiety Scale; HAMD, Hamilton Depression Scale; GAD-7, Generalized Anxiety Disorder7; PHQ-9, Patient Health Questionnaire-9; PHQ-15, Patient Health Questionnaire-15; JFLS, Jaw Functional Limitation Scale; OBC, Oral Behavior Checklist; VAS, Visual Analogue Score; NA, Not Available; TMD, Temporomandibular disorder; Num, Number; M, Male; F, Female; NC, healthy control. ^a^Median (the first quartile, the third quartile).*

### 3.2 Differences topological properties between TMDp group and NCs

The brain functional networks of subjects in both TMDp and NC groups had small-world properties within the sparsity threshold range of 0.05~0.20, that is, γ > 1, λ ≈ 1, σ > 1 (Fig. 1). As shown in Table 2, the Cp and Eloc of the two groups had significant differences when sparsity was 0.05 and 0.06 (Fig. 2). Eg and Lp had no statistical significance between 2 groups (*p * > 0.05) (Fig. 3).

**Fig. 1.** The curves of the small world attribute parameters of the brain function network with sparsity. grp 1: normal control group; grp 2: painful temporomandibular disorder group. (a) the standardized clustering coefficient; (b) the standardized shortest path length; (c) the small-world index. Red represents normal control group, and blue represents painful temporomandibular disorder group.

**Table 2.** Comparison of global attributes between TMDp group and NC group.

Topological properties (threshold)	TMD	NC	*t* value	*p* value
Cp (0.05)	0.276 ± 0.007	0.295 ± 0.007	1.839	0.0350
Cp (0.06)	0.322 ± 0.007	0.343 ± 0.008	1.742	0.0429
Eloc (0.05)	0.342 ± 0.007	0.363 ± 0.008	1.873	0.0326
Eloc (0.06)	0.402 ± 0.008	0.426 ± 0.101	1.667	0.0499

*Cp, clustering coefficient; Eloc, local efficiency; TMD, Temporomandibular disorder; NC, healthy control.*

**Fig. 2.** The curves of the clustering coefficient and the local efficiency of the brain function network with sparsity. *Indicates a statistically significant difference between the two groups at the threshold (*p * < 0.05).

**Fig. 3.** The curves of the global efficiency and the shortest path length of the brain function network with sparsity.

As shown in Table 3, Eloc of TMDp patients with a sparsity of 0.06 were positively correlated with HAMD and GAD-7 (*r* = 0.319, *p* = 0.042; *r* = 0.348, *p* = 0.026; respectively), while Eg, Lp, γ, λ and σ presented no significant correlations with the clinical variables.

**Table 3.** Correlation analysis between brain network topological properties with significant difference and clinical variables.

Topological properties		Cp (0.05)	Cp (0.06)	Eloc (0.05)	Eloc (0.06)
HAMA^a^					
	*r*	0.242	0.243	0.270	0.252
	*p*	0.128	0.127	0.088	0.111
HAMD^a^					
	*r*	0.185	0.268	0.232	0.319
	*p*	0.247	0.090	0.144	0.042
GAD-7^a^					
	*r*	0.200	0.288	0.289	0.348
	*p*	0.210	0.068	0.067	0.026
PHQ-9^a^					
	*r*	0.024	0.031	0.076	0.071
	*p*	0.880	0.850	0.635	0.660
PHQ-15^a^					
	*r*	0.058	0.166	0.092	0.206
	*p*	0.717	0.299	0.569	0.196
JFLS^b^					
	*r*	0.177	0.119	0.256	0.213
	*p*	0.269	0.459	0.107	0.181
OBC^b^					
	*r*	−0.011	0.049	0.043	0.103
	*p*	0.947	0.761	0.790	0.522
VAS^a^					
	*r*	0.067	0.113	0.078	0.169
	*p*	0.677	0.483	0.627	0.292

*HAMA, Hamilton Anxiety Scale; HAMD, Hamilton Depression Scale; GAD-7, Generalized Anxiety Disorder7; PHQ-9, Patient Health Questionnaire-9; PHQ-15, Patient Health Questionnaire-15; JFLS, Jaw Functional Limitation Scale; OBC, Oral Behavior Checklist; VAS, Visual Analogue Score; Cp, clustering coefficient; Eloc, local efficiency. ^a^Spearman; ^b^Pearson.*

It is worth noting that the multiple comparisons conducted in this study did not undergo weighted correction for family error rates. Therefore, the reported *p* values should be interpreted with caution, as the possibility of false positive results increases.

## 4. Discussion

Brain network can be categorized into structural network and functional network. The functional network, which is constructed based on the structural network, also reflects the functional roles of the brain’s structural network [32, 33]. This study used fMRI technique to investigate the topology attribute of the brain functional network in TMDp patients and NCs in resting state, aiming to reveal the alterations in cerebral function among TMDp patients. Both the brain networks of NCs and TMDp patients demonstrated small-world properties. As demonstrated in Fig. 1, both groups maintained fundamental small-world organization (σ > 1.5), yet the concurrent attenuation of γ, λ and σ values in TMD signifies a fundamental reorganization of functional brain architecture. This tripartite reduction reveals a novel network adaptation pattern characterized by three interdependent phenomena: First, decreased γ values indicate weakened local cluster cohesion, reflecting functional fragmentation within specialized modules. Second, as with the conventional interpretations of reduced λ for global efficiency gains, our data demonstrate accelerated information integration in TMD. Third, the attenuated small-world index (σ) denotes a shift toward an efficient-yet-fragile topological state, wherein heightened global efficiency (λ↓) facilitates rapid nociceptive signaling while diminished resilience (γ↓) manifests as vulnerability to network disruptions. Collectively, we observed a synchronous decline (triple decay) in the γ, λ and σ values of the TMD group, suggesting an adaptive pattern for the transition to an efficient-fragile network state: patients maintain cognitive function via optimized hub routing despite exhibiting pain-perpetuating hypersynchronization in nociceptive pathways. However, it must be emphasized that the key statistical tests supporting this model have not undergone family error rate correction for multiple comparisons. Future research requires the application of strict correction methods in independent samples for reproduction verification.

Notably, our results indicated that when the sparsity was set to 0.05 and 0.06, significant differences were observed in the clustering coefficient and local efficiency between the TMDp group and the NC group. Additionally, the Eloc values of TMDp patients showed a positive correlation with the scores of HAMD and GAD-7. Notably, our analysis revealed comparable topological configurations between TMDp patients and healthy controls across multiple network metrics—including path length characteristics, clustering coefficients, small-world organization, and global integration capacity.

The small-world properties of the brain network are one of the foundations for realizing rapid information exchange and integration between different brain regions, which facilitate information transmission and processing in the brain network to be carried out efficiently and with low energy consumption [34]. More importantly, by comparing the coefficients and indices of brain networks varied across different thresholds of correlation coefficients between TMDp group and NC group, we further observed alterations in the small-world topology of the brain network in TMDp patients. The clustering coefficient reflects the tendency of nodes in a network to form clusters. A higher Cp indicates that local nodes in the network are more tightly connected, implying stronger capacity for short-range information transmission. In the current study, the TMDp group exhibited a lower Cp when sparsity was set to 0.05 and 0.06, suggesting a weakened capacity of the network for local information processing. Numerous studies have demonstrated that the brain functional networks of both healthy subjects and patients with certain functional brain diseases possess small-world properties. Additionally, there were some changes in the small-world properties of patients with partly diseases such as Alzheimer’s disease, obsessive-compulsive disorder, and schizophrenia [35, 36, 37].

Previous studies have demonstrated that TMDp patients could present decreased functional connectivity in the rest-state brain network associated with pain perception and pain processing [27, 38], which is speculated to be related to the network communication interruption during the information processing. Given that the anterior insula and anterior cingulate cortex are key components of the cognitive-affective brain network involved in pain processing [39], and that the anterior brain regions of TMDp patients continuously receive information related to painful noxious stimuli, Ichesco [25] speculated that the increased functional connection strength between the anterior insula and anterior cingulate cortex plays an important role in emotional regulation and anti-nociception, which is an adaptive change in patients with TMDp. As TMD is characterized by painful motor dysfunction, He *et al*. [27] found that TMDp patients showed decreased functional connectivity in cortex-striatal circuits (ventral striatum and anterior cingulate, anterior insula, dorsal striatum and primary motor cortex) compared to healthy controls, which was associated with clinical symptom indicators including clinical dysfunction index and pain intensity.

Previous studies have shown that the striatum can participate in the motor response to pain [40, 41, 42], with its activation contributing to the regulation of orofacial pain [43, 44], while the anterior insula and anterior cingulate play an important role in pain regulation and cognitive processing. Therefore, the weakened cortico-striatal pathways and reduced functional connectivity within the striatum may be associated with impairments in motor control, pain processing, and cognition in TMD. In the present study, the decreased brain network connectivity may refer that the continuous chronic pain state can disrupt the interaction and balance state between the brain network, and the altered functional activity of the brain network may be associated with the endogenous and self-persistence of chronic pain in TMD patients. The current study also revealed the lower Cp in TMDp patients, indicating reduced connectivity and information transmission in pain-related brain networks. Above all, no significant differences were observed in γ, λ, or σ between the two groups, which may be attributed to the small sample size in this study.

Furthermore, when the sparsity was 0.05 and 0.06, TMDp patients showed decreased local efficiency of the functional brain networks, which was positively correlated with symptoms of anxiety and depression. In contrast, no significant decrease in global efficiency was observed in TMDp patients compared to NCs. Global efficiency quantifies information transfer across the entire network, while local efficiency assesses these capabilities at the nodal level, providing complementary perspectives on network integration [45, 46, 47, 48]. These findings suggest that in TMDp patients, the capacity and efficiency of information transmission in brain functional networks are impaired at the local level, whereas global efficiency of cerebral information transmission would not be significantly decreased. In addition, mounting evidence has implicated that TMDp patients had abnormal FC within default mode network (DMN) involved in cognition, emotion, and memory regulation and between the DMN and pain-related networks [28, 49, 50, 51] which could be supposed to be related to the decreased connectivity between nodes and the decreased efficiency to transmit information at close range, or long-term pain input in TMDp patients.

Additionally, recent findings have revealed that higher scores on HAMD and GAD-7 are associated with increased Eloc in TMDp patients, which suggested that the local processing capacity of the brain functional network enhances with the aggravation of negative emotions including anxiety and depression. In line with these findings, we speculate that the abnormalities in certain properties of the brain functional network, such as increased Eloc, may be one of the significant factors contributing to pain-related negative emotional symptoms in TMDp patients. Therefore, the current study may also provide potential insights and a new perspective for further understanding of the neuromechanisms of pain and the functional reorganization of the brain in TMDp patients.

This study has some limitations. Firstly, the sample size is small, and future research should further investigate how different types of TMD-related pain affect brain functional networks. Secondly, there remains a critical need for emotion-modulated fMRI examinations to characterize dysregulated cerebral activation under stressful emotional conditions. Thirdly, Notably, while the sparsity range (0.05–0.20) was predetermined based on established methodological standards, the specific thresholds demonstrating group differences (0.05 and 0.06) emerged from exploratory analysis within this range, which may impact the generalizability of these particular findings. Fourthly, network comparisons across sparsity thresholds were conducted as independent analyses; future studies may benefit from family-wise error correction approaches. Fifthly, the absence of significant group differences in anxiety and depression scores (HAMA/HAMD) may reflect recruitment constraints: while control participants were stringently screened to exclude psychiatric histories, TMD patients inherently presented with clinically relevant symptom profiles, potentially obscuring nuanced psychological distinctions between groups. Finally, although this study identified differences in the topological properties of brain networks, the specific brain regions associated with these differences in spatial distribution remain unclear, which will be a focus of our future research. And the node-level or regional network analysis would provide more specific insights into the brain regions that drive the observed topological changes.

## 5. Conclusions

In summary, the current study demonstrated that the decreased clustering coefficient and decreased local efficiency were identified in TMDp patients when the sparsity was 0.05 and 0.06, which suggested that the capability and efficiency of brain functional networks to process and transmit information may be impaired from the local level in TMDp, and may be the neuromechanism of the degree of reflection on negative emotions. Therefore, the topological properties of the brain network based on rs-fMRI could be considered as a new simple tool to monitor the dysfunction of the brain in TMDp.
